# Ethnic density and mortality: aboriginal population health in Taiwan

**DOI:** 10.1186/s40985-016-0028-1

**Published:** 2016-10-03

**Authors:** Shao-Chiu Juan, Tamara Awerbuch-Friedlander, Richard Levins

**Affiliations:** 1 School of Criminal Justice, State University of New York at Albany, 135 Western Avenue, Albany, 12222 NY USA; 2 Department of Global Health and Population, Harvard School of Public Health, 655 Huntington Avenue, Boston, 02115 MA USA

**Keywords:** Ethnic density, Mortality, Aboriginal population, Alcoholism, Health disparities

## Abstract

**Background:**

Ethnic density (the proportion of ethnic minority populations in a geographic area) has emerged as an important factor determining population health. By examining the relationship between mortality rates and the proportion of aboriginal population in Taiwan, this ecological approach highlights the pressing need to understand why aboriginal health remains relatively disadvantaged affecting the population as a whole, especially given the provision of universal health coverage.

**Methods:**

Using combined data from various government departments in Taiwan, we first compare overall mortality rates between aboriginal people and the general population in Taiwan’s 21 administrative locations during the years 2010 and 2011. Then we describe the associations between ethnic density and the relative risk of 40 different causes of death.

**Results:**

Aboriginal people in Taiwan on average have higher overall mortality rates than the general population. The proportion of aboriginal population is associated with a higher risk of death for overall mortality, homicide, vehicle crashes, tuberculosis, and several alcohol-related diseases such as peptic ulcer, chronic liver disease, and cirrhosis. These affect the health of the general population in counties where aborigines are abundant.

**Conclusion:**

The proportion of aboriginal population may play an essential role in determining Taiwan’s population health. When universal health coverage is in place, the root causes (for example, alcoholism, culture, and socioeconomic disadvantages) of health disparities between aboriginal populations and general populations need to be addressed.

## Background

### Ethnic density effects on mortality

While older studies showed that racial/ethnic minority groups might enjoy better health outcomes when living in locations with a higher density of their own groups because enhanced social networks, mutual support, and a stronger sense of belongingness acquired from one’s own group are likely to be positively associated with health [1, 2], more recent analyses have shown that the density of racial/ethnic minorities, in terms of residential segregation and ethnic enclave, is associated with the increased levels of deprivation, which contribute to worse health outcomes [3]. Across different nations, aboriginal populations show a general pattern of socioeconomic disadvantage [4, 5].

### Aboriginal people in Taiwan

Taiwanese aborigines account for only 2.2 % of 23.4 million people living in Taiwan [6]. Compared to Han Chinese populations—the majority of racial/ethnic group which began to migrate from Mainland China in the seventeenth century—aboriginal people in Taiwan had experienced economic competition and military conflicts with a series of colonizing newcomers. As a result, Taiwanese aborigines were forced to live in concentrated villages along high mountains, which further restrict their access to socioeconomic resources; and it is not until the early 1980s that many aboriginal groups started to actively seek better economic development and political self-determination [7]. Nowadays, aborigines reside in both the mountains and major cities, especially Hualien and Taitung which are known for larger proportions of aboriginal communities [6]. Despite genetic evidence that intermarriage has contributed to 88 % of Taiwanese populations who carry some degree of aboriginal origin [8], substantial cultural differences in terms of language, social customs, and lifestyle still place aboriginal communities in a relatively disadvantaged position [9].

### Health disparities between aboriginal people and the general population

Similar to aboriginal groups in Australia, the USA, and other countries [4, 10], Taiwanese aborigines also suffer from alcoholism and other poorer health outcomes when compared to the general population [11, 12], and such gaps in health status between Taiwanese aborigines and Han Chinese seem to increase over time. For example, an epidemiological study examining 993 participants from four Taiwanese aboriginal groups found that the lifetime prevalence of alcoholism meeting the clinical criteria of International Classification of Diseases (ICD-10) and the third Diagnostic and Statistical Manual of Mental Disorders (DSM-III-R) was up to 54.7 and 55.5 %, respectively, much higher than the rates (0.11 to 0.16 %) reported 40 years ago [12]. Despite a lack of similar estimates in Taiwan, research in Australia informs that alcoholism accounts for at least 10 % of all deaths among Australian aboriginal populations [10]. Despite the fact that Taiwan’s National Health Insurance (NHI), launched in 1995, has covered over 99 % of total population with increased average life expectancy by 2012 [13], an earlier study (1994) examining aboriginal health in Taiwan found that the average life expectancy of aborigines is 10 years less than that of the general population, 12.5 years less for men, and 6 years less for women [14]. Recent official statistics (2011) shows that aborigines’ life expectancy is much shorter: 8.6 years less than that of the general population (70.6 vs. 79.2 years), almost 10 years less for men (66.1 vs. 76), and 7.3 years less for women (75.3 vs. 82.6) [15, 16]. Obviously, health disparities persist over time. When comparing aboriginal women and total female populations, the gaps in life expectancy even increased from 6 to 7.3 years.

As such, there is a pressing need to examine cause-specific mortality rates through which health policy can be developed to address certain causes linked to Taiwanese aborigines’ high mortality rates and whether these causes vary with ethnic density. To our best knowledge, this is the first study to examine the relationship between ethnic density and mortality in Taiwan.

## Methods

We conducted an ecological analysis to examine the association between ethnic density and mortality. The data consist of two parts: (1) health statistics: overall and cause-specific mortality rates of the total population (i.e., aboriginal population + non-aboriginal population) [17] and overall mortality rates of the aboriginal population from the year 2010 through 2011 [18]; and (2) ethnic density: the proportion of aboriginal population reported in December 2010 for Taiwan’s 21 administrative units [19]. Ethnic density, by definition, is the proportion of ethnic minority populations in a geographic area [3]. Mortality rates are calculated as the average number of deaths per 100,000 people and per year. Forty cause-specific mortality rates for the general population are classified by the tenth version of the International Classification of Diseases (ICD-10). However, we do not have access to aboriginal cause-specific mortality rates because these statistics are not publicly available. To ensure data compatibility across multiple government departments, the study period is limited to the years 2010 and 2011 because geographical units and diagnostic codes have been changed in earlier years. We exclude one administrative unit—Matzu island for its smallest population size (less than 0.1 % of total population) and missing information in national datasets. Ethnic density is calculated as the number of aboriginal people divided by the number of total residents in a given administrative location. Correlation analyses are used to describe the relationship between ethnic density and mortality rates.

## Results

### Overall mortality rates: aboriginal population versus general population

As shown at the bottom row of Table 1, aboriginal people are at higher risk for death compared to the general population in Taiwan. The annual, average overall mortality rate of Taiwanese aborigines is 14 % higher than that of the general population during the years 2010 and 2011 (728.87 vs. 640.64 per 100,000 people).

**Table 1:** Ratio of overall mortality rate, aboriginal population versus general population, Taiwan, 2010–2011

Administrative locations	Proportion of aboriginal population (%)	Mortality rates		Mortality rate ratio (aboriginal/general)
		Aboriginal	General	
Taitung County	34.75	1108.52	992.53	1.12
Hualien County	26.84	998.03	914.54	1.09
Pingtung County	6.52	931.39	869.14	1.07
Nantou County	5.37	852.89	834.49	1.02
Hsinchu County	3.81	887.20	662.94	1.34
Ilan County	3.36	747.19	740.41	1.01
Taoyuan County	2.96	421.44	522.94	0.81
Keelung City	2.26	420.22	689.86	0.61
Miaoli County	1.90	855.58	784.80	1.09
New Taipei City	1.25	186.31	486.14	0.38
Kaohsiung City	1.07	443.27	660.22	0.67
Taichung City	1.05	246.33	549.63	0.45
Chiayi County	1.01	875.59	918.83	0.95
Hsinchu City	0.72	668.90	541.72	1.23
Taipei City	0.52	254.64	589.61	0.43
Kinmen County	0.50	15082.64	526.89	28.63
Changhua County	0.38	283.11	696.14	0.41
Chiayi City	0.32	344.04	633.83	0.54
Tainan City	0.31	223.06	723.16	0.31
Penghu County^a^	0.31	0.00	904.89	0.00
Yunlin County	0.25	310.03	885.11	0.35
Total	2.21	728.87	640.64	1.14

^a^Mortality rate of aboriginal people in Penghu County is zero, most likely due to missing information; these official statistics were drawn from death reports and may be subject to reporting errors

### Ethnic density and the ratios of overall mortality rates

The second column of Table 1 is ordered by the proportion of aboriginal population in a given location from the highest to the lowest. As mentioned earlier, Taitung County and Hualien County are well-known for their aboriginal communities. Consistent with overall mortality rates for the whole nation, overall mortality rates of aborigines living in these two counties are also higher than those of the general population (1108.52 vs. 992.53 in Taitung; 998.03 vs. 914.54 in Hualien). To examine whether such pattern of relationship also applies to ethnicity density, we performed correlation analyses and found that the proportion of aboriginal population is positively correlated (Pearson’s correlation coefficient *ρ* = 0.74) with overall mortality rates for total populations. This correlation remains large in magnitude (*ρ* = 0.59) after removing two influential data points (two offshore islands as Kinmen and Penghu Counties); non-parametric test (Kendall’s correlation) also shows positive correlations (tau-a = 0.23; tau-b = 0.23 after adjusting for ties). In other words, people residing in locations with a higher proportion of aboriginal people tend to have a higher risk of death. Additionally, the proportion of aboriginal population is also positively correlated with overall mortality rates for aboriginal populations (*ρ* = 0.64; tau-a = 0.57; tau-b = 0.57; excluding Kinmen and Penghu Counties). Consistent with newer analyses which imply the negative effect of ethnic density [3], our study findings show that a higher concentration of aboriginal population, in terms of overall mortality rates, does not reflect better health for both the general and aboriginal populations.

### Ethnic density and cause-specific mortality rates

Table 2 shows that ethnic density is positively associated with certain causes of death, especially homicide (*ρ* = 0.85), vehicle crashes (*ρ* = 0.77), tuberculosis (*ρ* = 0.81), and several alcohol-related diseases such as peptic ulcer (*ρ* = 0.85) and chronic liver diseases and cirrhosis (*ρ* = 0.77). Parallel to previous findings that aboriginal people on average have a shorter life span than others [14–16], our study shows that ethnic density is negatively associated with senility (*ρ* = −0.15), possibly indicating that due to a constellation of multiple factors (e.g., dietary or cultural habits, socioeconomic disadvantages, and other confounders), people living in locations with a higher proportion of aboriginal people on average are less likely to enjoy a longer life expectancy, which might also explain why aging-related diseases such as dementia are more frequently reported among non-aboriginal populations in certain countries [20].

**Table 2:** Correlation between the proportion of aboriginal population and cause-specific mortality rates, Taiwan, 2010–2011

	Cause-specific mortality rates	Correlation coefficient
1	Intestinal infectious diseases	−0.17
2	Tuberculosis***	0.81
3	Septicemia	0.24
4	Viral hepatitis	0.11
5	Human immunodeficiency virus (HIV) disease	0.13
6	Malignant neoplasms	0.29
7	Remainder of neoplasms	0.09
8	Anemias*	0.43
9	Diabetes mellitus	0.28
10	Vascular and unspecified dementia	−0.19
11	Meningitis**	0.60
12	Spinal muscular atrophy and related syndromes	0.18
13	Parkinson’s disease	−0.17
14	Alzheimer’s disease	0.02
15	Hypertensive diseases**	0.60
16	Diseases of heart (except hypertensive diseases)**	0.63
17	Cerebrovascular diseases	0.54
18	Atherosclerosis*	0.49
19	Aortic aneurysm and dissection	−0.16
20	Influenza	−0.09
21	Pneumonia*	0.45
22	Acute bronchitis and bronchiolitis**	0.51
23	Chronic lower respiratory diseases**	0.58
24	Pneumoconioses	−0.10
25	Lung diseases due to external agents (except pneumoconiosis and pneumonia)	0.04
26	Peptic ulcer***	0.85
27	Hernia and intestinal obstruction**	0.59
28	Chronic liver disease and cirrhosis***	0.77
29	Cholelithiasis and other disorders of gallbladder*	0.32
30	Diseases of the skin and subcutaneous tissue	0.23
31	Diseases of the musculoskeletal system and connective tissue**	0.59
32	Nephritis, nephrotic syndrome, and nephrosis	0.24
33	Pregnancy, childbirth, and the puerperium	−0.19
34	Certain conditions originating in the perinatal period*	0.37
35	Congenital malformations, deformations, and chromosomal abnormalities*	0.43
36	Senility	−0.15
37	Sudden infant death syndrome	0.16
38	Vehicle crashes***	0.77
39	Intentional self-harm (suicide)*	0.43
40	Assault (homicide)***	0.85

Notes: The magnitude of correlation: *0.3–0.5 moderate effect; **0.5–0.7, large effect; ***0.7–0.9, very large effect

## Conclusion

While the implementation of universal health coverage has once rendered Taiwan the second healthiest country of the world [21], health disparities between aboriginal people and general population in Taiwan has persisted over the past decade and still persists. At a population level, we find that Taiwanese people living in areas with a higher density of aboriginal people are likely to have higher overall mortality risk and more likely to die from homicides, vehicle crashes, tuberculosis, and alcohol-related diseases. That is, ethnic density may play an important role in determining Taiwan’s population health.

Our study makes it clear that provision of universal healthcare is not a panacea for health disparities in Taiwan and does not effectively reduce the mortality risk among aboriginal populations. Existing literature has highlighted that in addition to inadequate healthcare resources, social disadvantages also contribute to aborigines’ poorer health than Han Chinese [4]; similar underlying causes of health inequalities such as social exclusion, stress, and addictions have also been reported among aboriginal populations in other nations [22, 23]. While mainstream research puts the spotlight on health disparities between White, Black, and Latino populations in the USA, empirical and ecological studies examining aboriginal populations in the USA, New Zealand, and Canada clearly indicate that, when compared to the general population, aboriginal people are at higher risk of deaths, especially deaths due to motor vehicle crashes, suicide, and homicide; this risk might be related to aboriginal groups’ poverty, marginalization, and social disorganization [4], which has also been noticed in Australia’s population studies [5]. Due to data availability and the small sample size, however, our study cannot fully address these associations. Although ecological research can be very important for hypothesis generation or reporting overall trends, it alone cannot support causality claims.

While reformers of healthcare system strive to shrink the gap in health outcome between better-off and vulnerable populations, the consequences of socioeconomic inequality based on the proportion of aboriginal population should not be ignored. We hope that our study, using Taiwan as an example, can prompt public health experts and policy makers to identify, intervene, and eventually alleviate the root causes of health disparities.

## Abbreviations

DSM: Diagnostic and Statistical Manual of Mental Disorders
ICD: International Classification of Diseases
NHI: National Health Insurance
